# Allopurinol attenuates acute kidney injury following *Bothrops jararaca* envenomation

**DOI:** 10.1371/journal.pntd.0006024

**Published:** 2017-11-20

**Authors:** Pedro Henrique França Gois, Monique Silva Martines, Daniela Ferreira, Rildo Volpini, Daniele Canale, Ceila Malaque, Renato Crajoinas, Adriana Castello Costa Girardi, Maria Heloisa Massola Shimizu, Antonio Carlos Seguro

**Affiliations:** 1 Laboratory of Medical Research–LIM12, Nephrology Department, University of Sao Paulo School of Medicine, Sao Paulo, Brazil; 2 Royal Brisbane and Women’s Hospital, Nephrology Department, Brisbane, Australia; 3 Vital Brazil Hospital, Butantan Institute, Sao Paulo, Brazil; 4 Heart Institute (InCor), University of Sao Paulo School of Medicine, Sao Paulo, Brazil; Texas A&M University Kingsville, UNITED STATES

## Abstract

Snakebites have been recognized as a neglected public health problem in several tropical and subtropical countries. *Bothrops* snakebites frequently complicate with acute kidney injury (AKI) with relevant morbidity and mortality. To date, the only treatment available for *Bothrops* envenomation is the intravenous administration of antivenom despite its several limitations. Therefore, the study of novel therapies in *Bothrops* envenomation is compelling. The aim of this study was to evaluate the protective effect of Allopurinol (Allo) in an experimental model of *Bothrops jararaca* venom (BJ)-associated AKI. Five groups of Wistar rats were studied: Sham, Allo, BJ, BJ+Allo, BJ+ipAllo. BJ (0.25 mg/kg) was intravenously injected during 40’. Saline at same dose and infusion rate was administered to Sham and Allo groups. Allo and BJ+Allo groups received Allo (300 mg/L) in the drinking water 7 days prior to Saline or BJ infusion respectively. BJ+ipAllo rats received intraperitoneal Allo (25 mg/Kg) 40’ after BJ infusion. BJ rats showed markedly reduced glomerular filtration rate (GFR, inulin clearance) associated with intense renal vasoconstriction, hemolysis, hemoglobinuria, reduced glutathione and increased systemic and renal markers of nitro-oxidative stress (Nitrotyrosine). Allo ameliorated GFR, renal blood flow (RBF), renal vascular resistance and arterial lactate levels. In addition, Allo was associated with increased serum glutathione as well as reduced levels of plasma and renal Nitrotyrosine. Our data show that Allo attenuated BJ-associated AKI, reduced oxidative stress, improved renal hemodynamics and organ perfusion. It might represent a novel adjuvant approach for *Bothrops* envenomation, a new use for an old and widely available drug.

## Author summary

Snakebites have been recognized as a neglected public health problem in several tropical and subtropical countries. Snakes from the genus *Bothrops* are the most common snakes found in Latin America. *Bothrops* snakebites frequently complicate with kidney impairment with increased risk of death. The only treatment currently available for *Bothrops* envenomation is the infusion of antivenom. Therefore, the study of novel therapies to prevent kidney failure caused by snakebites is compelling. Allopurinol is a drug commonly used to treat gout which also presents beneficial antioxidant properties. The aim of this study was to evaluate the protective effect of Allopurinol in a rat experimental model of *Bothrops* envenomation. In our study, rats that received the infusion of the snake venom presented with reduced kidney function which was improved by Allopurinol. Overall, Allopurinol might represent a novel approach to prevent kidney failure in victims of *Bothrops* snakebites. This could be a new use for an old and widely available drug.

## Introduction

Snakebites have been recognized as a neglected public health problem in several tropical and subtropical countries [1]. The worldwide incidence of snake envenomation may vary from 421,000 to 1,841,000 cases each year, resulting in up to 94,000 deaths [2]. However, the burden of snakebite envenomation might be underestimated, since many incidence data are derived from hospital admissions and most of the affected individuals do not seek hospital treatment [3].

Despite the nearly universal distribution, venomous snakebites are mainly found among populations living in poverty as it is inversely related to the health care expenditure [4]. These accidents predominantly affect young adults, especially males, yet almost 1/3 of the cases occur in children [5]. Moreover, snake envenomation may also be seen as an occupational hazard whereas it has been associated with significant morbidity and disability mostly in agricultural workers (food producers) [4–6]. Thus, there is a great impact of venomous snakebites in the economy of a community or even a country.

Snakes of the genus *Bothrops* are the major cause of snakebites in Latin America [5]. Over 60 species distributed throughout Central and South America and the Caribbean Basin constitute the genus *Bothrops* [7,8]. In Latin America, *Bothrops asper*, *Bothrops atrox* and *Bothrops jararaca* are the main medically important *Bothrops* species [5,9]. Previous studies have reported substantial differences in venom composition among the genus *Bothrops* which might be explained by variations within species, geographic regions, dietary availability, or even by age difference considering the same animal [8,10–16]. However, some venom biological properties are common to all *Bothrops* species [8].

*Bothrops* venoms possess many active enzymes responsible for the local and systemic manifestations [7]. Local edema is a common finding which may be associated with drastic hemorrhagic and necrotic changes [17]. Furthermore, *Bothrops* venoms exhibit thrombin-like enzyme activity which activates the coagulation cascade and ultimately causes fibrinogen consumption, bleeding and disseminate intravascular coagulation [5,8,18]. Bothrops envenomation frequently complicates with acute kidney injury (AKI) increasing morbidity and mortality [10,19].

To date, the only treatment available for *Bothrops* envenomation is the intravenous administration of antivenom (animal hyperimmune immunoglobulin). However, there are several limitations of the antivenom therapy such as high cost, limited availability, (e.g. in remote areas), severe anaphylactic reactions, and inefficiency to treat local manifestations [6,20–22]. Therefore, the study of novel therapies in *Bothrops* envenomation is a fruitful area for future research.

Allopurinol (Allo) is a xanthine oxidase (XO) inhibitor commonly used as uric acid (UA)-lowering agent. Data from several studies suggest that beneficial effects of Allo as a free radical scavenger may transcend the XO inhibition such as reducing lipid peroxidation and heat shock protein expression [23,24]. Additionally, treatment with Allo following rhabdomyolysis-associated AKI showed renal and muscular protective effects by reducing oxidative stress and UA levels [23]. Thus, new therapeutic applications have been accredited to Allo in the last decade particularly due to its potent antioxidant property [23,25–27]. Strapazzon et al. showed marked and persistent increase of several oxidative stress markers in patients hospitalized for *Bothrops* envenomation [28]. Therefore, the aim of this study was to evaluate the protective effect of Allo, a low-cost and widely available medication, in an experimental model of Bothrops jararaca venom (BJ)-associated AKI.

## Materials and methods

### Ethics statement

All experimental procedures were approved by the local research ethics committee (“Comissão de Ética no Uso de Animais”–CEUA, 070/17) and developed in strict conformity with local institutional guidelines and with well-established national standards for the manipulation and care of laboratory animals (“Resoluções Normativas do Conselho Nacional de Controle de Experimentação Animal”–CONCEA). Animals were anesthetized intraperitoneally with sodium thiopental (50 mg/kg BW) for all the surgical experiments. After the experimental period, animals were euthanized with excess anesthetic sodium thiopental.

### *Bothrops jararaca* venom

A pool of lyophilized venom from specimens of adult *B*. *jararaca* snakes was a generous gift from the Institute Butantan, São Paulo, Brazil (lot 01/08–01). *B*. *jararaca* snakes are kept in plastic cages in temperature and humidity-controlled rooms, according the Institute Butantan Animal Care and Use Committee (protocol n° 1296/16). The venom is extracted monthly and after centrifugation the supernatant is frozen. Subsequently venoms are pooled, lyophilized, and stored at *−*20°C until used. On the experiment day, aliquots were dissolved in 10 ml of 0.9% saline for immediate use.

### Allopurinol

In the present study, we aimed to use doses of Allo well established in other experimental rat studies [29–31]. Nevertheless, in view of the U.S. Food and Drug Administration (FDA) approach to make a dose extrapolation from humans to rats, the dose of a rat would be approximately seven times higher than the human dose (per Kg of BW) [32]. Thus, taking into consideration a daily dose of Allo (300 mg) in an adult human weighting 60 kg (5 mg/Kg/day), the dose of Allo to be administered to the rat would be approximately 35 mg/kg/day. Considering the mean daily water intake of 25 mL/rat/day and mean BW of 272 g, we used a dose of approximately 28 mg/kg/day in the BJ+Allo group. BJ+ipAllo animals received 25 mg/Kg BW of Allo, which was around 5 times the human dose.

### Animals and experimental protocol

Male Wistar rats with a mean BW of 272±5 g were obtained from the animal facilities of the University of São Paulo School of Medicine, housed in standard cages, on a 12h light/dark schedules, and given *ad libitum* access to water and standard diet (Nuvilab CR-1, Curitiba-PR, Brazil). Room temperature was maintained at 23°C.

We administered intravenous BJ after the surgical procedure for the inulin clearance study, as previously reported by Martines et al. [6]. Animals were allocated into four groups:

1. Sham (control) (n = 5): received 1.6 ml of 0.9% saline via the right jugular vein for 40 min at a rate of 0.02 ml/min (Compact Infusion Pump, 975, Harvard, USA);
2. Allo (n = 5): received Allo in the drinking water (300 mg/L of water) for seven days before inulin clearance studies. Animals also received 0.9% saline infusion as the Sham group;
3. BJ (n = 6): received 0.25 mg/kg of BJ via the right jugular vein for 40 min at a rate of 0.02 ml/min;
4. BJ+Allo (n = 5): received Allo in the drinking water (300 mg/L of water) for seven days prior to BJ infusion above described.

**Fig 1:**
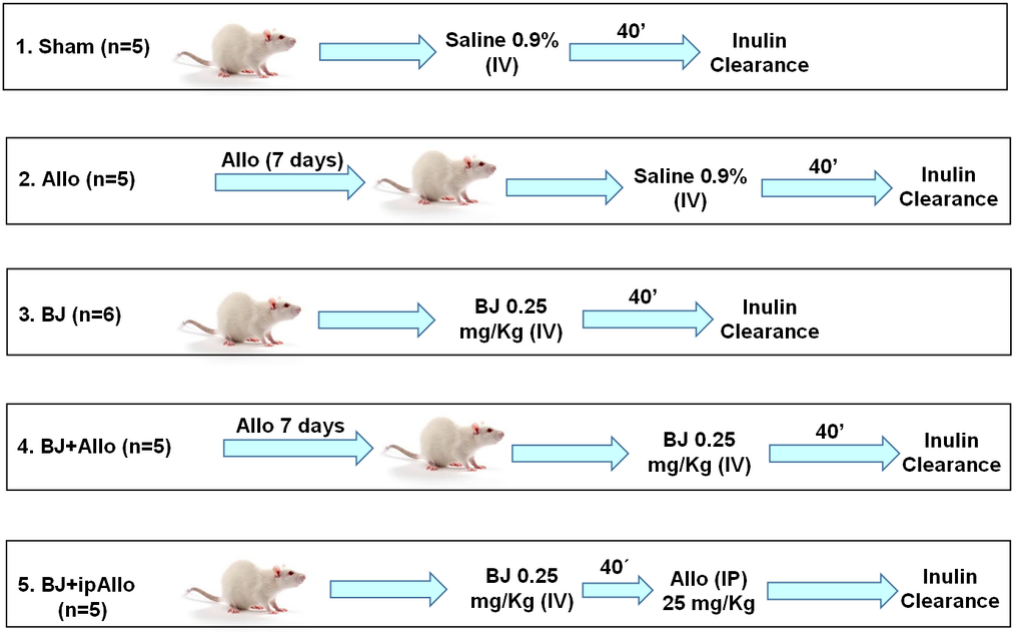
Experimental protocol. Five groups were studied: Sham (n = 5), Allopurinol (Allo) (n = 5), *Bothrops jararaca* (BJ) (n = 6), BJ+Allo (n = 5), BJ+ipAllo (n = 6). BJ (0.25 mg/kg) was injected intravenously during 40’. Saline at same dose and infusion rate was administered to Sham and Allo groups. Allo and BJ+Allo groups received Allo (300 mg/L) in the drinking water 7 days prior to Saline/BJ infusion. BJ+ipAllo rats received intraperitoneal Allo (25 mg/Kg) 40’ after BJ infusion.

In order to study the therapeutic effect of Allo on renal function, we performed an additional group of animals (BJ+ipAllo, n = 5) that received intraperitoneal Allo (Sigma-Aldrich, St. Louis, USA) (25 mg/kg BW) immediately after BJ infusion. Fig 1 illustrates the experimental protocol in the first series of experiments.

In separate series of experiments, using the same experimental model described above, we conducted hemodynamic studies after intravenous BJ or saline infusion (five groups of six rats/group). Animals were anesthetized intraperitoneally with sodium thiopental (50 mg/kg BW). The trachea was cannulated with a PE-240 catheter, and spontaneous breathing was maintained. To evaluate the mean arterial pressure (MAP) and allow blood sampling, a PE-60 catheter was inserted into the right carotid artery. MAP was measured 30 minutes after the surgical procedure, using Biopac Systems Inc MP100 (Santa Barbara, CA, USA). Then, a midline incision was made to measure the renal blood flow (RBF). We carefully dissected the left renal pedicle and isolated the renal artery, taking precautions to avoid disturbing the renal nerves. An ultrasonic flow probe was placed around the exposed renal artery. RBF was measured using an ultrasonic flow meter (T402; Transonic Systems, Bethesda, MD, USA) and is expressed as ml/min. Renal vascular resistance (RVR) was calculated by dividing the blood pressure by RBF and is expressed as mmHg/ml/min.

### Determination of renal function

To determine the glomerular filtration rate (GFR), we conducted inulin clearance studies. On the day of the experiment, animals were anesthetized and cannulated as above. For infusion of inulin and fluids, another PE-60 catheter was inserted into the left jugular vein. For BJ or 0.9% saline infusion, a PE-60 catheter was inserted into the right jugular vein. In order to collect urine samples, a suprapubic incision was made, and the urinary bladder was cannulated with a PE-240 catheter. After surgical procedure, a loading dose of inulin (100 mg/kg BW diluted in 0.9% saline) was administered through the jugular vein. Subsequently, a constant inulin infusion (10 mg/kg BW in 0.9% saline) was started and was continued at 0.04 ml/minute throughout the experiment. Blood samples and three urine samples were obtained at the beginning and at the end of the experiment. Blood and urine inulin were determined using the anthrone method. GFR data are expressed as ml/min/100g BW.

### Renal tissue sample collection and preparation

At the end of either inulin clearance, organs were perfused with PBS solution (0.15 M NaCl and 0.01 M sodium phosphate buffer, pH 7.4). The right kidney was removed and frozen in liquid nitrogen and stored at −80°C. The left kidney was cleaned of connective tissue and fixed in 10% formalin.

### Total protein isolation

Kidney sections were homogenized in an ice-cold isolation solution (200 mmol/l mannitol, 80 mmol/l HEPES, 41 mmol/l KOH, pH 7.5) containing a protease inhibitor cocktail (Sigma, St. Louis, MO, USA), using a Teflon-pestle glass homogenizer (Schmidt and Co., Frankfurt am Main, Germany). The homogenates were centrifuged at low speed (4,000 rpm) for 30 min at 4°C to remove nuclei and cell debris. Pellets were suspended in isolation solution with protease inhibitors. Protein concentrations were determined using the Bradford assay method (Bio-Rad Protein Assay Kit; Bio-Rad Laboratories, Hercules, CA, USA).

### Oxidative stress studies

Plasma and renal nitrotyrosine (NT) levels, which are stable end products of peroxynitrite oxidation, were measured using a commercial ELISA kit (HK: 501–02; Hycult Biotech, Uden, the Netherlands).

Reduced GSH, the major endogenous antioxidant in cells, was determined in total blood by the method of Sedlak and Lindsay [33]. Whole blood was processed by addition of four volumes of ice-cold 5% metaphosphoric acid and centrifuged at 4,000 rpm for 10 min at 4°C. This assay consists of the reaction of supernatants of total blood samples with Ellman’s reagent to produce a yellow pigment measured spectrophotometrically at 412 nm. Serum GSH was quantified by means of the standard curve and reported as μmol/mL.

### Biochemical assessments

Plasma sodium and potassium were measured by flame photometry (CELM, model FC280, São Paulo, SP, Brazil). Hematocrit, serum lactate and bicarbonate were determined with specific electrodes (ABL800Flex–Radiometer, Brønshøj, Denmark). Fibrinogen was assessed using the Clauss modified method (hemostasis coagulation analyzer, Stago Start 4, France). Lactate dehydrogenase (LDH) was determined with a kinetic method using UV absorbency, which measures the conversion from L-lactate in pyruvate (automatized analyzer, Cobas C111, Roche, Switzerland). The enzymatic colorimetric method (Labtest, Lagoa Santa, Brazil) was used to measure plasma creatine phosphokinase (_P_CPK) and plasma uric acid (UA). Hemoglobinuria was assessed using a dipstick urine test (Cobas, Roche, Switzerland).

### Light microscopy

Four-micrometer histological sections of kidney tissue were stained with hematoxylin–eosin (HE) or Masson’s trichome and examined under light microscopy. For histomorphometry, the images obtained by microscopy were captured on video via an image analyzer (Axiovision; Carl Zeiss, Eching, Germany). We analyzed 30 grid fields (0.087 mm^2^ each) per kidney cortex. The interstitial areas were demarcated manually, and the proportion of the field they occupied, excluding the glomeruli, was determined. In 40–60 grid fields (0.245 mm^2^ each; magnification, x400), we graded the proportional renal damage (tubular epithelial swelling, vacuolar degeneration, necrosis, and desquamation): 0, < 5%; I, 5–25%; II, 26–50%; III, 51–75%; and IV, > 75%. To minimize bias in the morphometric analysis, the observer was blinded to the treatment groups. The mean scores were calculated by rat and by group.

### Immunohistochemistry

We used monoclonal antibody to 8-isoprostane-PGF_2a_ (F2-IsoP) (1:500, overnight at 4°C) (Oxford Biomedical Research, Oxford, England). We subjected 4-μm kidney tissue sections to immunohistochemical reaction according to the protocol for the primary antibody. Reaction products were detected by an avidin-biotin-peroxidase complex (Vector Laboratories, Burlingame, CA), and the color reaction was developed with 3,3-diaminobenzidine (Sigma Chemical) in the presence of hydrogen peroxide. The sections were counterstained with Harris’ hematoxylin. To evaluate immunoreactivity to F2-IsoP, we analyzed 15 randomly fields of the renal medulla (0.087 mm^2^ each). The volume ratios of positive areas of renal medula (%), determined by the color limit, were obtained by image analysis with the program Image-Pro Plus, version 4.1 (Media Cybernetics, Silver Spring, MD) on a computer coupled to a microscope (Axioskop 40; Carl Zeiss) and a digital camera. Results were expressed as percentages.

### Statistical analysis

Data are shown as mean ± SEM. Differences among the means of multiple parameters were analyzed by one-way analysis of variance followed by the Student–Newman–Keuls test. Values of P<0.05 were considered statistically significant. Data were analyzed using GraphPad Prism software 5.0.

## Results

### Allo pretreatment attenuated *Bothrops jararaca* venom-associated AKI

**Fig 2:**
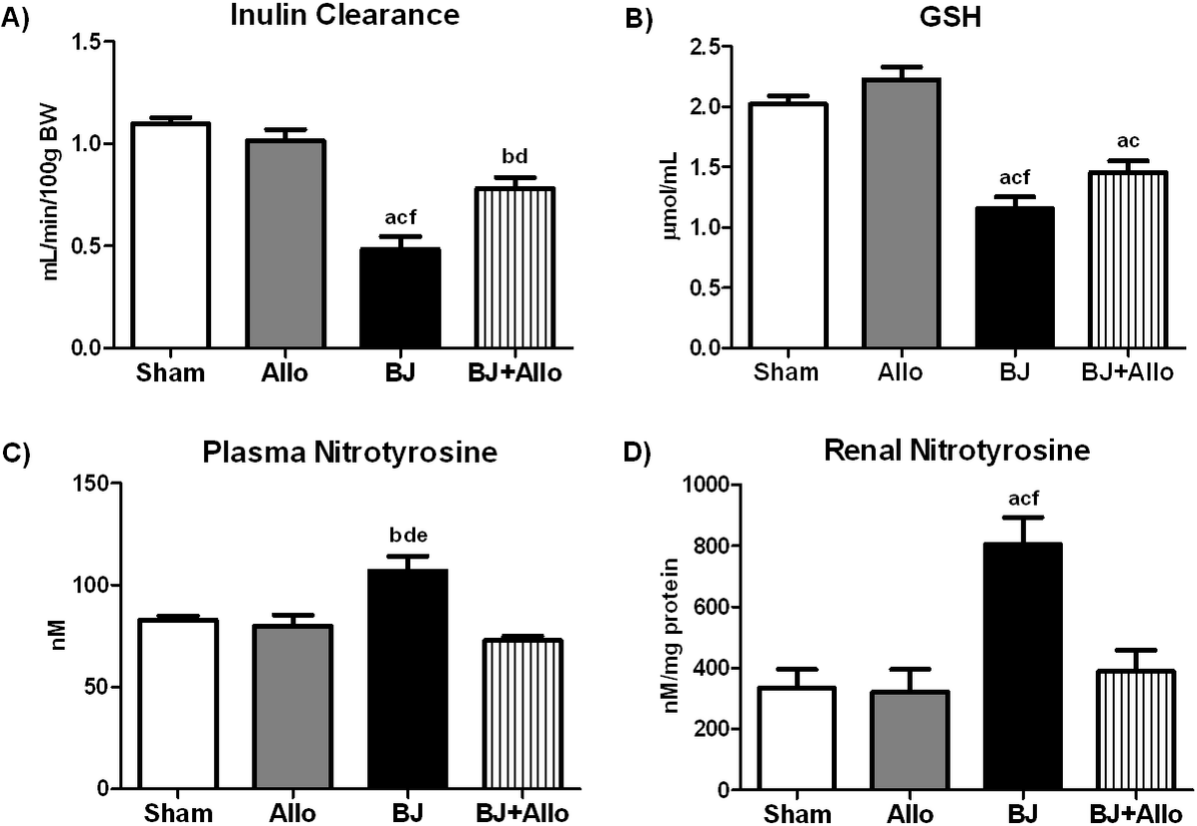
Allopurinol (Allo) attenuates renal dysfunction and improves redox balance. A) Inulin clearance studies: gold standard assessment of renal function. B) Serum reduced glutathione (GSH). C) Plasma Nitrotyrosine and D) Renal Nitrotyrosine. Data are mean ± SEM; Sham (n = 5): control; Allo (n = 5): Allo pretreatment; BJ (n = 6): *Bothrops jararaca* venom infusion; BJ+Allo (n = 5): BJ+Allo pretreatment ^a^p<0.001, ^b^p<0.05 vs. Sham; ^c^p<0.001, ^d^p<0.05 vs. Allo; ^e^p<0.001, ^f^p<0.05 vs. BJ+Allo.

To evaluate the efficacy of prophylactic Allo on BJ-associated AKI, animals received Allo seven days prior to intravenous venom infusion. Renal function, assessed by inulin clearance, was markedly reduced in the BJ group compared to controls. In contrast, Allo pretreated rats exhibited significantly better renal function (Fig 2A).

**Fig 3:**
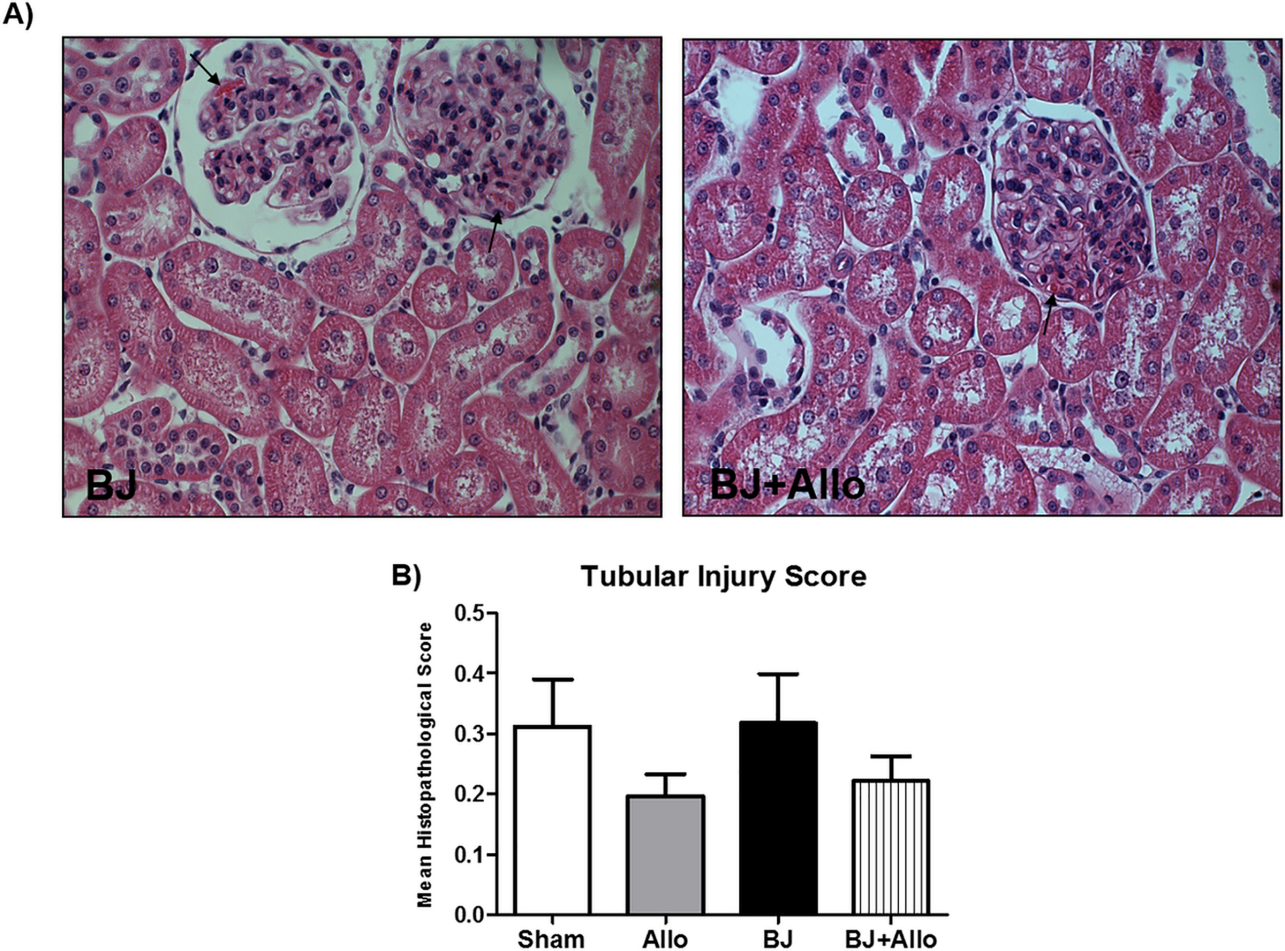
Histopathology of renal cortex. A) Representative photomicrographs of kidney tissue samples from BJ and BJ+Allo groups. Arrows: glomerular microthrombi deposit. Magnification, x400. Hematoxylin–eosin. B) Bar graph of tubular injury score.

Histopathological examination of kidney tissues showed glomerular microthrombi deposit in the groups that received BJ (Fig 3A). Sham and Allo groups did not show any significant histopathological changes. Tubular injury score was not different between groups (Fig 3B).

### Allo pretreatment decreased systemic and renal oxidative stress in *Bothrops jararaca* venom-associated AKI

To assess whether the potent antioxidant effect of Allo would play a role in renal protection in the current experimental model, we evaluated serum reduced GSH (a major endogenous antioxidant), plasma and renal NT (a marker of marker of nitro-oxidative stress). Animals from the BJ group exhibited lower levels of serum reduced GSH compared to control animals. Allo pretreatment improved serum reduced GSH levels (Fig 2B). On the other hand, BJ rats exhibited higher levels of plasma and renal NT, which returned to control levels in the group pretreated with Allo (Fig 2C and 2D). Overall, these data suggest an improvement in the systemic and renal redox balance conferred by Allo following *B*. *jararaca* envenomation.

### Allo pretreatment improved renal blood flow in *Bothrops jararaca* venom-associated AKI

**Fig 4:**
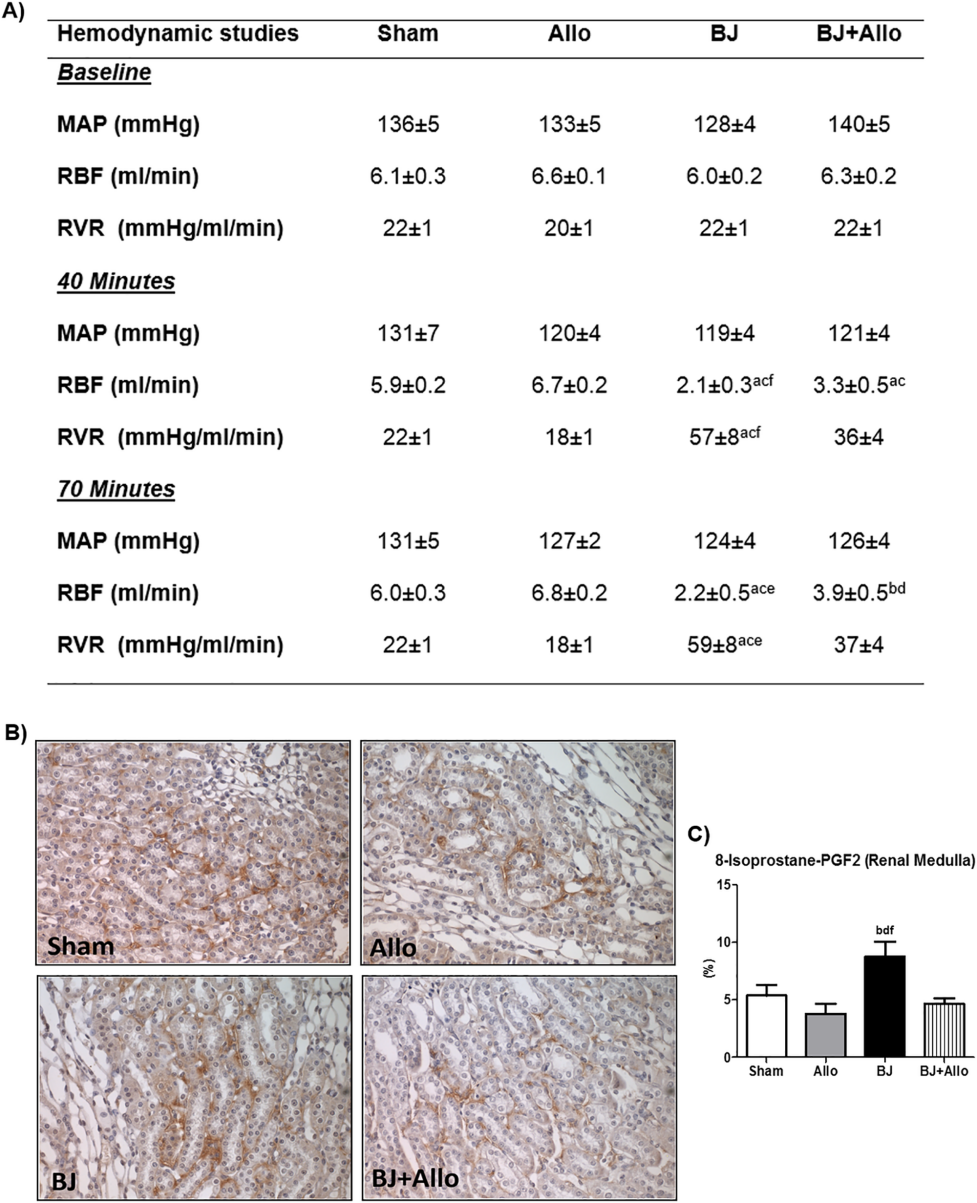
Allopurinol (Allo) restored renal blood flow. A) Hemodynamic studies at baseline, 40 and 70 minutes. Mean arterial pressure (MAP), renal blood flow (RBF) and renal vascular resistance (RVR). B) Immunostaining (brown) for F2-IsoP in the renal medulla. Magnification, x400. C) Bar graph of F2-IsoP renal medulla staining quantification. Data are mean ± SEM; Sham (n = 6): control; Allo (n = 6): Allo pretreatment; BJ (n = 6): Bothrops jararaca venom infusion; BJ+Allo (n = 6): BJ+Allo pretreatment ^a^p<0.001, ^b^p<0.05 vs. Sham; ^c^p<0.001, ^d^p<0.05 vs. Allo; ^e^p<0.001, ^f^p<0.05 vs. BJ+Allo.

Renal ischemia has been considered the hallmark of AKI following *B*. *jararaca* envenomation [6,34,35]. Accordingly, we observed severe renal vasoconstriction, associated with increased renal vascular resistance, in BJ animals at 40 and 70 minutes after venom infusion. Allo pretreatment significantly improved RBF and RVR at both time points evaluated. MAP was lower in the BJ group after BJ infusion, albeit not statistically significant. Fig 4A shows a summary of the hemodynamic data.

In addition, we found increased expression of F2-IsoP, a potent vasoconstrictor formed during lipid peroxidation, in the renal medulla (Fig 4B). BJ animals showed significant increased expression of F2-IsoP in the renal medulla compared to other groups. Allo pretreatment reduced F2-IsoP medullary renal expression (Fig 4B).

### Allo pretreatment on other systemic effects of *Bothrops jararaca* venom

**Fig 5:**
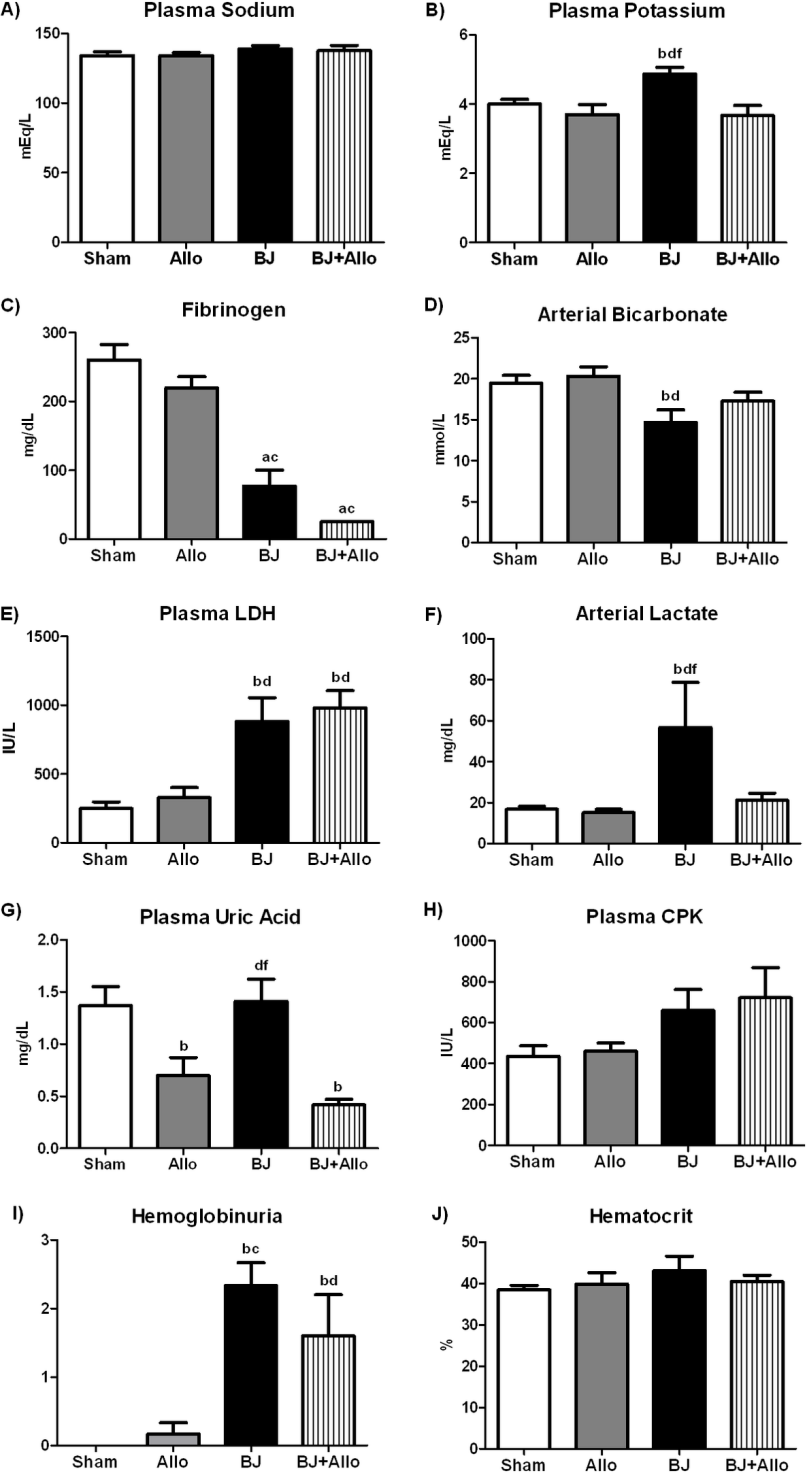
Allopurinol (Allo) on other systemic parameters following *Bothrops jararaca* envenomation. A) Plasma sodium. B) Plasma potassium. C) Fibrinogen. D) Arterial bicarbonate. E) Plasma lactate dehydrogenase (LDH). F) Arterial lactate. G) Plasma uric acid. H) Plasma creatine phosphokinase (CPK). I) Hemoglobinuria (Dipstick: 0: no hemoglobinuria; 1: +; 2: ++; 3: +++). J) Hematocrit. Data are mean ± SEM; Sham (n = 5): control; Allo (n = 5): Allo pretreatment; BJ (n = 6): *Bothrops jararaca* venom infusion; BJ+Allo (n = 5): BJ+Allo pretreatment ^a^p<0.001, ^b^p<0.05 vs. Sham; ^c^p<0.001, ^d^p<0.05 vs. Allo; ^e^p<0.001, ^f^p<0.05 vs. BJ+Allo.

Plasma sodium was not different between groups, whereas animals from BJ group showed higher plasma potassium compared to other groups (Fig 5A and 5B). Furthermore, BJ infusion led to a marked decrease in fibrinogen and bicarbonate levels whilst increased serum LDH and lactate levels. BJ+Allo rats exhibited lower arterial lactate, yet fibrinogen, LDH and bicarbonate changes were not prevented by Allo pretreatment (Fig 5C–5F). Moreover, Allo-pretreated rats showed lower plasma UA concentration (Fig 5G). As expected, _P_CPK was slightly increased, albeit not statistically significant, after intravenous BJ administration (Fig 5H). Although we found hemoglobinuria in animals that received BJ, there were no differences on hematocrit levels between groups (Fig 5I and 5J).

### Allo treatment attenuated *Bothrops jararaca* venom-associated AKI and improved renal hemodynamics

We have conducted additional studies using intraperitoneal Allo after BJ infusion in order to assess if therapeutic Allo also improved the functional parameters (renal function, RBF and RVR). Strikingly, therapeutic Allo also improved renal function (0.74 ± 0.05 mL/min/100g BW; p<0.01 vs. BJ group), RBF (4.4 ± 0.5 mL/min; p<0.01 vs. BJ group) and RVR (18.5 ± 1.5 mmHg/mL/min; p<0.001 vs. BJ group) following BJ envenomation.

## Discussion

AKI seems to be a major cause of death among patients who survived the early BJ-induced systemic effects [10]. Animal models of AKI following intravenous infusion of BJ have been validated to study the mechanisms involved in this syndrome as well as potential therapeutic agents [6,34,35]. Similar to previous studies, we found that intravenous administration of BJ caused striking reduction of renal function associated with intense renal vasoconstriction, fibrinogen consumption and intravascular hemolysis without systemic blood pressure changes. We also reported that prophylactic and therapeutic Allo attenuated renal dysfunction secondary to BJ infusion. To our knowledge, this is the first demonstration of Allo as both treatment and prevention of BJ-associated AKI.

In recent years, there has been an increasing amount of literature on the role of oxidative stress in the pathogenesis of snake envenomation [28,36,37]. Snake venoms present a series of biologically active peptides, such as L-amino acid oxidase (LAAO) and phospholipase A2 (PLA2) which might be related to the generation of reactive oxygen species (ROS) [38,39]. LAAOs are flavoenzymes widely distributed among venomous snake families that catalyze the oxidative deamination of L-amino acid to α-keto acids, thus, releasing hydrogen peroxide and ammonia [39]. Venom PLA2, in contrast to endogenous PLA2, has been linked with a wide spectrum of toxic effects, leading to increased systemic oxidative stress [39,40]. Therefore, to evaluate the involvement of the oxidative stress in the pathogenesis of BJ-associated AKI, as well as its inhibition by Allo therapy, we assessed serum reduced GSH, plasma and renal NT. GSH is the most prevalent intracellular thiol, whose main role is to protect cells from oxidative damage [41]. On the other hand, NT is a byproduct of protein tyrosine residues generated from the reaction of nitric oxide (NO) and superoxide (O2^•—^) [42]. In recent years, it has been demonstrated the important role of NO regulating the redox signaling [43]. When reacted with O2^•—^, NO is a major substrate to generate reactive nitrogen species which can lead to nitration and nitrosation of several substrates, mostly proteins with various physiological functions [43]. This biological process has been called by some authors as nitro-oxidative stress [43–45]. Furthermore, Walker et al. described renal NT as an early marker of oxidative stress in the setting of renal ischemia reperfusion [46]. We found evidence of redox imbalance in the rats from BJ group. BJ infusion was associated with reduced levels of GSH levels and increased systemic and renal markers of nitro-oxidative stress, whilst Allo pretreatment improved all these markers.

Renal vasoconstriction is one of the key elements in the pathogenesis of BJ-associated AKI [6,34,35]. Martines et al. showed a significant reduction of RBF and increase of RVR following BJ infusion [6]. Furthermore, previous studies have highlighted the pivotal role of isoprostanes in oxidative stress-mediated renal vasoconstriction [23,47,48]. Accordingly, we found profound renal vasoconstriction combined with increased expression of F2-IsoP in the renal medulla. We believe that two factors might have contributed to this finding. First, given the high sensitivity of the medulla to decreased O2 supply, it is reasonable to expect that it would be the first portion of the kidney affected by vasoconstriction and oxidative stress [49]. Second, since we performed an early assessment of F2-IsoP (90’ after BJ infusion), we might not have been able to detect cortical alterations at this stage. Allo ameliorated renal hemodynamic changes in this experimental model. We suggest that Allo might have inhibited the lipid peroxidation and consequently reduced F2-IsoP formation. Conversely, F2-IsoP may represent a marker of increased oxidative stress following intense vasoconstriction in the renal medulla. Nevertheless, we could not provide evidence of causality between F2-IsoP and BJ-induced renal vasoconstriction and this is still an important issue for future research.

In addition to the renal hemodynamic changes, fibrin thrombi deposits in the glomerular capillary may also play a role in the pathogenesis of BJ-associated AKI contributing to the reduction in GFR and RBF [34]. Hence, data from necropsy kidney samples showed glomerular fibrin thrombi following *Bothrops* snakebites in individuals with cortical necrosis [50]. We showed glomerular microthrombi deposit in the groups that received BJ. Nevertheless, tubular injury score was not different between groups, which might be due to the renal tissue harvesting in the very early stage of AKI, therefore, too early to observe significant renal histological damage. On the other hand, we did not find significant difference in CPK levels among the study groups, which speaks against rhabdomyolysis as a pathogenic factor for AKI in this experimental model. In fact, Burdmann et al. also reported no increase in CPK levels using similar animal model. We believe that rhabdomyolysis is mainly a local manifestation of BJ envenomation, thus, occurring after subcutaneous or intramuscular inoculation of the venom rather than with intravenous injection.

In the past years, numerous studies have reported the pro-oxidant properties of UA [51–53]. Higher UA levels may activate NADPH oxidase, increasing protein nitrosation and lipid peroxidation [54]. Moreover, several lines of evidence suggest an association between UA with hypertension, cardiovascular disease, progression of chronic kidney disease and rhabdomyolysis-associated AKI [23,55–57]. In the present study, we assume that the positive effects of Allo resulted predominantly from free radical scavenging. However, we may not neglect the potential adjuvant effect of UA-lowering therapy. Therefore, further research is warranted to uncover the role of UA in the pathogenesis of BJ-associated AKI.

*Bothrops* venoms are composed of several different substances related to broad range of systemic effects [7]. Intravenous infusion of BJ has been linked with fibrinogen consumption, intravascular hemolysis and shock (reduced organ perfusion). Similarly, our animal model reproduced all the systemic effects of BJ envenomation as described in previous studies [6,34,35]. However, one limitation of our experimental model was the short follow up after BJ infusion. This might be the reason why hemoglobin levels as well as MAP were not significantly lower in the BJ group compared to other groups. In fact, we believe that we might have observed a period of cryptic shock (hyperlactatemia without persistent hypotension) associated with initial features of hemolysis (increased LDH and hemoglobinuria) and hemoconcentration, the latter also described in early stages of hemorrhagic shock [58]. Overall, BJ+Allo rats showed better organ perfusion (i.e. lower arterial lactate levels) which is in accordance with previous studies that reported protective effects of Allo in experimental models of hemorrhagic shock by improving hemodynamics, preserving tissue levels of ATP and ultimately reducing mortality [59,60]. Therefore, the studies discussed here highlighted the role of Allo beyond free radical scavenging in the setting of shock.

In conclusion, our study show that Allo attenuated BJ-associated AKI, reduced oxidative stress, improved renal hemodynamics and organ perfusion. We hope that this study encourages further clinical investigation regarding the beneficial effects of Allo on *Bothrops* envenomation. If supported by clinical studies, it might represent a novel adjuvant approach for *Bothrops* envenoming, a new use for an old and widely available drug.
